# Cloning and molecular characterization of *ant(9)-If*: a novel aminoglycoside adenylyltransferase gene conferring resistance to spectinomycin

**DOI:** 10.3389/fmicb.2026.1776824

**Published:** 2026-04-07

**Authors:** Susu Chen, Huiqin Liu, Jie Yang, Yali Huang, Xiuxiu Wang, Junjun Wang, Lulu Huang, Xiaolan Wu, Junwan Lu, Qiyu Bao, You Zhou, Teng Xu, Hongqin Zhang

**Affiliations:** 1 Department of Laboratory Sciences, Pingyang Hospital of Wenzhou Medical University, Wenzhou, China; 2 Institute of Translational Medicine, Baotou Central Hospital, Baotou, China; 3 Institute of Biomedical Informatics/School of Laboratory Medicine and Life Sciences, Wenzhou Medical University, Wenzhou, China; 4 Medical Molecular Biology Laboratory, School of Medicine, Jinhua University of Vocational Technology, Jinhua, China

**Keywords:** adenylyltransferase, *ant(9)-If*, *Enterococcus faecium*, kinetic parameter, resistance gene

## Abstract

**Background:**

*Enterococcus* is an opportunistic pathogen with the potential to induce infections in both animals and humans. The antimicrobial resistance mechanism of *Enterococcus* is highly complex, leading to challenges in the clinical treatment of infectious diseases caused by this pathogen. Consequently, a better understanding of antibiotic resistance mechanisms is urgently needed.

**Methods:**

Sewage samples were obtained from animal farms, and the streaking plate technique was used to isolate single colonies. The whole-genome sequence of the bacterium was obtained using next-generation sequencing platforms. Antimicrobial minimum inhibitory concentrations (MICs) were determined using the standard agar dilution method. Molecular cloning was performed to explore the function of the antibiotic resistance gene. The protein was expressed, and its enzyme kinetic parameters were analyzed. Bioinformatic methods were used to examine the sequence structure and evolutionary dynamics of the resistance gene.

**Results:**

In this study, a novel plasmid-encoded aminoglycoside resistance gene, *ant(9)-If*, was discovered in *Enterococcus faecium* 520 isolated from animal farm sewage. Compared with the control strain JH2-2/pAM401, the recombinant strain JH2-2/pAM401-*ant(9)-If* demonstrated a 32-fold increase in the MIC of spectinomycin. ANT(9)-If shares 64.70% amino acid identity with the drug resistance enzyme ANT(9)-Ia and demonstrated high affinity and catalytic efficiency for spectinomycin, characterized by a Michaelis constant (*K*_*m*_) of 4.599 ± 0.198 μM and a *k*_*cat*_/*K*_*m*_ value of (8.78 ± 0.39) × 10^4^ M^–1^⋅s^–1^. Sequence analysis revealed that the novel resistance gene *ant(9)-If* is associated with a mobile genetic element (MGE) encoded on the plasmid. The *ant(9)-If*(*-*like) genes are broadly distributed across plasmids and chromosomes in various Gram-positive cocci and bacilli, including those of clinically significant species.

**Conclusion:**

In contrast to *ant(9)-Ic* and *ant(9)-Id*, which were identified from the chromosomes of Gram-negative bacilli, the novel aminoglycoside resistance gene *ant(9)-If*, associated with the MGE encoded on the plasmid, was identified in a Gram-positive *coccus* strain isolated from animal farm sewage. Deeper exploration of the antimicrobial resistance mechanisms of bacteria from various sources can aid in the development of effective treatments for infections in both animal husbandry and human healthcare. These studies could expedite the development of strategies to prevent the spread of resistance among bacteria from various sources.

## Introduction

Enterococci, a group of Gram-positive bacteria, are ubiquitously present in the environment and digestive tracts of both humans and animals. As an opportunistic pathogen, *Enterococcus* can infect major food-producing animals, such as pigs, cattle, sheep, ducks, and chickens (Cebeci, 2024). Furthermore, it can lead to human septicemia, endocarditis, meningitis, urinary tract infections, skin and soft-tissue infections, and abdominal infections (Fiore et al., 2019). In recent years, with the increasing incidence and mortality associated with human enterococcal infections, enterococci have been designated as the second major pathogen causing nosocomial infections in the United States Hospital Infection Surveillance System (NISS) (Weiner-Lastinger et al., 2020). According to data from the National Antibiotic Resistance Monitoring System (NARMS) in the United States, the prevalence of Enterococcus in livestock and poultry meat samples is the highest compared to that in other samples (Marshall and Levy, 2011). With the invention and utilization of antibiotics, particularly the extensive application of antibiotics in animal husbandry, the prevalence of drug-resistant *Enterococcus* has been continuously increasing, and the scope of available antibiotic options has been gradually narrowing. In addition to its intrinsic resistance to some commonly used clinical antibiotics, such as β-lactams, *Enterococcus* can also acquire resistance to macrolides, glycopeptides, tetracyclines, etc., through MGEs and other mechanisms. This resistance poses significant challenges to the clinical treatment of bacterial infections (Hollenbeck and Rice, 2012). Studies have reported that antimicrobial resistance can spread horizontally among enterococci from different sources, including those from humans and animals (Choi and Woo, 2015). Consequently, research on enterococcal drug resistance is no longer confined to a single field but encompasses public health, agriculture, and animal husbandry. Therefore, a more in-depth understanding of the mechanisms of *Enterococcus* drug resistance is urgently needed to effectively treat and control the diseases caused by *Enterococcus*.

Aminoglycosides are potent broad-spectrum antibiotics used to treat bacterial infections. They inhibit bacterial protein synthesis to exert antibacterial effects and have been used to treat life-threatening infections (Serio et al., 2018). Currently, the mechanisms of bacterial resistance to aminoglycoside antibiotics can be classified into the following categories: (1) aminoglycoside-modifying enzymes (AMEs) that chemically modify the drug structure, rendering it inactive; (2) mutations in the bacterial target sites corresponding to antibacterial agents; (3) alterations in bacterial membrane permeability to drugs; and (4) the overexpression and activation of bacterial efflux pumps. Although numerous resistance mechanisms exist, the most clinically relevant mechanisms are mediated by 16S rRNA methylases and three groups of antibiotic modification enzymes, namely, aminoglycoside adenylyltransferases (ANTs), aminoglycoside acetyltransferases (AACs), and aminoglycoside phosphotransferases (APHs) (Ramirez and Tolmasky, 2010; Garneau-Tsodikova and Labby, 2016). At present, approximately 80 types of aminoglycoside-modifying enzymes have been discovered. One enzyme often affects the activity of different antibiotics, and one antibiotic can also be modified by multiple modifying enzymes (Ramirez and Tolmasky, 2010). As a type of aminoglycoside antibiotic, spectinomycin can bind to the 30S ribosomal subunit of bacteria, thereby inhibiting protein translation and exerting antibacterial effects (Kanchugal and Selmer, 2020). Drug inactivation through adenylylation is the most prevalent mechanism of spectinomycin resistance, and many ANTs with a spectinomycin-resistant phenotype have been identified to date (Zárate et al., 2018). Five ANT subgroups have been identified: ANT(2”), ANT(4’), ANT(6), ANT(3”) and ANT(9). ANT(3”)s are the most common ANTs, and the genes encoding them are often referred to as *aadA*. Many of these genes are associated with integrons and transposons and are carried by plasmids, facilitating their extensive dissemination (Sheng et al., 2023). Several genotypes of *ant(9)*, such as *ant(9)-Ia* and *ant(9)-Ib*, have been documented (Werner et al., 2013). The *ant(9)-Ia* gene was first identified in *Staphylococcus aureus* and later in *Enterococcus* species (Murphy, 1985). The *ant(9)-Ib* gene was identified to be encoded on a plasmid in *Escherichia coli* (LeBlanc et al., 1991). Among the aminoglycoside-modifying enzymes, the ANT(3”), ANT(9), and APH(9) proteins confer resistance to spectinomycin (Stern et al., 2018; Liang et al., 2021; Shi et al., 2023). Another typical mechanism of spectinomycin resistance involves mutations in chromosomal protein-coding or ribosomal RNA genes, resulting in corresponding changes in the ribosomal 30S subunit (Borovinskaya et al., 2007), and mutations in the 34-helix spectinomycin-binding region of 16S rRNA, leading to high-level spectinomycin resistance (Wilson, 2014).

Through molecular cloning, antimicrobial susceptibility testing, and whole-genome sequencing, a novel aminoglycoside-modifying enzyme-encoding gene, *ant(9)-If*, was identified and characterized in this study. The dynamic properties of the enzyme encoded by *ant(9)-If* were also analyzed.

## Materials and methods

### Bacterial strains and plasmids

The isolate *Enterococcus faecium* 520 was isolated from a sewage sample collected from an animal farm in Wenzhou, Zhejiang Province, China. The sample was inoculated onto a blood agar plate and incubated at 35°C for 24 h. Individual colonies were randomly selected. Phenotypically distinct colonies were first identified using the Vitek-60 automated microbial analysis system (bioMérieux, Inc., Craponne, France). Species identification of the isolate was confirmed using 16S rRNA gene homology, whole-genome average nucleotide identity (ANI) (Konstantinidis and Tiedje, 2005; Richter and Rosselló-Móra, 2009), and digital DNA–DNA hybridization (dDDH) analyses (Auch et al., 2010). The strains and plasmids employed in this study are presented in Table 1.

**TABLE 1:** Bacteria and plasmids used in this work.

Strains or plasmids	Relevant characteristics	Reference
*E. faecium* 520	An original isolate	This study
*Enterococcu*s *faecalis* JH2-2 (JH2-2)	A host for cloning the *ant(9)-If* gene	Our laboratory collection
*E. coli* BL21 (BL21)	A host for expressing ANT(9)-If	Our laboratory collection
*E. coli* ATCC 25922	A quality control strain for the antimicrobial susceptibility test	Our laboratory collection
pAM401-*ant(9)-If*/JH2-2	JH2-2 carrying the recombinant plasmid pAM401-*ant(9)-If*	This study
pCold I-*ant(9)-If*/BL21	BL21 carrying the recombinant plasmid pCold I-*ant(9)-If*	This study
pAM401	Cloning vector for the PCR products of the *ant(9)-If* gene with its upstream promoter region, AMP^r^	Our laboratory collection
pCold I	Expression vector for the PCR products of the open reading frame (ORF) of the *ant(9)-If* gene, AMP^r^	Our laboratory collection

^r^, resistance; AMP, ampicillin.

### Antimicrobial susceptibility test

In accordance with the CLSI M100,^1^ the agar dilution method was used to determine the MICs of antimicrobial agents against the bacteria. Antimicrobial stock solutions were serially diluted twofold in molten Mueller-Hinton agar (MHA) to yield certain concentration ranges (Table 2), and the final inoculum density of the bacterial suspensions was adjusted to ∼10^6^ CFU/mL. All agar plates were incubated at 37°C in an aerobic atmosphere (with 5% CO_2_) for 18–24 h. *E. coli* ATCC 25922 served as a quality control strain for antimicrobial susceptibility assays. The antimicrobial agents tested in this work included amikacin, gentamicin, streptomycin, tobramycin, kanamycin, spectinomycin, paromomycin, neomycin, netilmicin, and ribomycin.

**TABLE 2:** MIC test results (mg/L) using the agar dilution method for the recombinant and control strains.

Bacterium	GEN	TOB	SM	KAN	SPT	PAR	NEO	AMK	NET	RST
Range	1–128	1–128	1–1024	0.25–128	2–2048	0.25–128	0.25–128	1–1024	1–128	1–1024
*E. faecium* 520	16	32	**≥** 2048	128	128	> 128	>128	256	8	256
JH2-2	16	64	256	128	64	> 128	>128	512	8	256
pAM401/JH2-2	16	64	256	128	64	> 128	>128	512	8	256
pAM401-*ant(9)-If*/JH2-2	16	64	256	128	> 2048	>128	> 128	512	8	256

GEN, gentamicin; TOB, tobramycin; SM, streptomycin; KAN, kanamycin; SPT, spectinomycin; PAR, paromomycin; NEO, neomycin; AMK, amikacin; NET, netilmicin; RST, ribomycin.

### Genome sequencing and annotation

The genomic DNA of *E. faecium* 520 was subjected to sequencing utilizing the Illumina HiSeq 2500 and PacBio RS II platforms by Shanghai Personal Biotechnology Co., Ltd. (Shanghai, China). Long PacBio reads were assembled using the Unicycler program v0.5.0 (Wick et al., 2021), and the sequence quality was subsequently rectified with Illumina sequencing data using the pilon approach (Walker et al., 2014).

The program Prokka v1.14.6 (Seemann, 2014) was used to predict the open reading frames (ORFs), and DIAMOND v2.0.11 (Buchfink et al., 2021) was used to annotate the functions of the ORFs against the NCBI non-redundant protein database. Antimicrobial resistance genes were identified using Resistance Gene Identifier v5.2.02 and the Comprehensive Antibiotic Resistance Database (CARD) (McArthur et al., 2013). The ANI was determined using fastANI v1.33 (Jain et al., 2018), whereas dDDH was assessed using the Type Strain Genome Server (TYGS) online platform (Gilchrist and Chooi, 2021; Lian et al., 2021). The genetic context of *ant(9)-If* and its homologous genes was examined using clinker v0.0.24 (Gilchrist and Chooi, 2021). A multiple sequence alignment diagram and a neighbor-joining phylogenetic tree were constructed using MAFFT v7.490 and MEGAX software, respectively (Katoh and Standley, 2013; Kumar et al., 2018). The molecular weight and isoelectric point (pI) of ANT(9)-If were predicted using a JavaScript program.^2^

### Cloning of the resistance gene

*E. faecium* strain 520 genomic DNA was extracted using a Genomic DNA Miniprep Kit (Shanghai Generay Biotech Co., Ltd., Shanghai, China). For functional analysis of the predicted resistance gene, the target gene with its promoter region was amplified using polymerase chain reaction (PCR) with the primers listed in Table 3. The PCR products were inserted into the cloning vector pAM401 using T4 DNA ligase (Takara Bio, Inc., Dalian, China). The recombinant plasmid was transformed into JH2-2 cells using the calcium chloride method (Sambrook and Russell, 2006). The transformants were subsequently screened on LB agar plates supplemented with 16 μg/mL chloramphenicol, after which the cloned fragment was confirmed by sequencing.

**TABLE 3:** Primers used to clone the *ant(9)-If* gene.

Primer^a^	Sequence (5′–3′)^b^	Restriction endonuclease	Vector	Annealing temperature (°C)	Amplicon size (bp)
*ant(9)-If*-520-F	CGCTCTAGACAAAAATGGATAGACAGCAGTTTTGTAGC	/	pAM401	54°C	1,060
*ant(9)-If*-520-R	CGCGGATCCAAACTGATATATGGGGGAACATATCGAA	/		54°C	
ANT(9)-If-520-F	CGCGGATCCCTGGTGCCGCGCGGCAGCAGTATAGATTT AAGTAACAAAAAAATTCCGAAAGA	*Bam*HI + Thrombin	pCold I	63°C	807
ANT(9)-If-520-R	CCCAAGCTTTTAAACTATTCGAAAAGGTAATTGGATATT	*Hin*dIII		60°C	

*^a^*Primers beginning with *ant(9)* were utilized to amplify the ORF of *ant(9)-If* along with its promoter region. Primers beginning with ANT(9) were used to amplify the ORF of *ant(9)-If*.

*^b^*The underlined sequences indicate the restriction endonuclease/thrombin sites.

To express the protein, the open reading frame (ORF) of the *ant(9)-If* gene was obtained by PCR using the primers listed in Table 3. The PCR product was subsequently cloned and inserted into the pCold I vector between the *Bam*HI and *Hin*dIII restriction sites (Qing et al., 2004). The resulting recombinant plasmid, pCold I-*ant(9)-If*, was introduced into *E. coli* BL21 competent cells. The transformant (BL21/pCold I-*ant(9)-If*) was selected on LB agar plates supplemented with 100 μg/mL ampicillin, and the cloned fragment was subsequently confirmed by sequencing.

### Expression and purification of recombinant protein ANT(9)-If

The recombinant (BL21/pCold I-*ant(9)-If*) was cultured overnight in LB broth supplemented with ampicillin (100 μg/mL). The culture was then diluted 100-fold in fresh LB broth and incubated at 37°C to induce protein expression. ANT(9)-If expression was induced by the addition of 0.5 mM IPTG (isopropyl-β-D-thiogalactopyranoside) (Sigma Chemicals Co., St. Louis, MO, United States) when the optical density at 600 nm (OD_600_) of the culture reached 0.6–0.8, followed by further incubation at 15°C for 24 h. Bacteria were then harvested by centrifugation at 8,000 × g for 10 min and lysed via ultrasonication for 2 min. The supernatant was subsequently collected. The His-tagged target protein in the supernatant was purified using a Beyotime protein purification kit (Shanghai, China). The samples were incubated with recombinant enterokinase at 25°C for 2 h to cleave the His-tag. The purity of the ANT(9)-If protein was confirmed by SDS-PAGE using a 12% acrylamide gel and Coomassie blue G-250 staining, and its concentration was measured with a BCA protein assay kit (Beyotime, Shanghai, China).

### Kinetic studies of the ANT(9)-If enzyme

The kinetic parameters of purified ANT(9)-If toward spectinomycin were assessed using high-performance liquid chromatography (HPLC) on a Thermo Scientific AcceLA system (Thermo Fisher Scientific, Inc., China) at 37°C with a reaction volume of 100 μL. The 100 μL reaction system included 50 mM Tris-HCl (pH 7.5), 10 mM MgCl_2_, 0.1 mM ATP (pH 7.5), 6.06 × 10^–5^ μM purified ANT(9)-If protein, and varying concentrations of spectinomycin (0, 2, 3, 4, 5, 8, and 10 μM), with each concentration added in 10 μL. After the reaction system was preheated for 5 min, the assay was conducted on the analysis system. The mobile phases consisted of a mixture comprising KH_2_PO_4_ (100 mM), 5% acetonitrile, and 10% methanol. Non-linear regression of the steady-state kinetic parameters *k*_*cat*_ and *K*_*m*_ against the initial reaction rate was performed using the Michaelis–Menten equation in Prism (version 8.0.2; GraphPad Software, CA, United States). The value denotes the mean of three independent measurements.

### The GenBank accession numbers

The accession numbers for the *E. faecium* 520 chromosome, plasmid pEF520-244 and *ant(9)-If* gene were NZ_CM130009.1, NZ_JBRYYC010000002.1 and PX485637, respectively.

## Results and discussion

### Discovery of a novel aminoglycoside resistance gene

Approximately 200 isolates were obtained from sewage and soil samples obtained from domestic animal farms in Wenzhou, China, to explore the novel antimicrobial resistance mechanisms of environmental bacteria. Following antimicrobial susceptibility testing, draft genomes were sequenced using the Illumina sequencing platform, and numerous potential resistance genes for various categories of antimicrobials were annotated. Several hypothetical aminoglycoside resistance genes, such as *aph(6)-Ic*, *ant(9)-Ia*, *aac(2’)-IIb, aac(3)-IIIb*, *aac(6’)-Iaa*, and *aac(6’)-If* homologs, which exhibited less than 80.0% amino acid (aa) sequence similarity with the functionally characterized aminoglycoside resistance genes, were randomly selected and cloned. One of these genes, an *ant(9)-Ia* homolog designated *ant(9)-If* in this study, was identified as a functional aminoglycoside resistance gene.

Compared with the control JH2-2/pAM401 strain, the recombinant strain carrying *ant(9)-If* (JH2-2/pAM401-*ant(9)-If*) showed resistance to spectinomycin, with a 32-fold increase in the MIC. No variations in the MICs of the other antimicrobials tested were observed between the two strains (Table 2). The high MIC of spectinomycin in the recombinant strain might be partly attributed to the high expression level of *ant(9)-If*, which resulted from the high copy number of the cloning vector pAM401. The strain expressing *ant(9)-If* shared the same resistance spectrum as the strains expressing the other *ant(9)-I* genes. Upon analyzing the MICs of spectinomycin between strains expressing *ant(9)-If* and other *ant(9)-I* genes, the MIC of spectinomycin in the strain expressing *ant(9)-If* was > 2-fold higher than that in the strain expressing *ant(9)-Ic* (Sheng et al., 2023), but ≤ 2-fold lower than that in the strain expressing *ant(9)-Ib* (LeBlanc et al., 1991) or the strain expressing *ant(9)-Id* (Yu et al., 2024).

### General characteristics of the *E. faecium* 520 genome

The novel resistance gene *ant(9)-If* was encoded in an isolate named 520, which was sourced from animal farm sewage. Species classification of the isolate revealed that its 16S rRNA gene sequence was 100% similar (coverage × identity) to those of *Enterococcus faecium* E7114 (LR135443.1) and *Enterococcus faecium* SRR24 (CP038996.1). The isolate also had the highest ANI (98.7%) and dDDH (93.2%) compared with those of the type strain *E. faecium* SRR24. The ANI is widely recognized as the gold standard for classifying prokaryotic species (Jain et al., 2018). According to this standard, an isolate could be assigned to a particular species when its ANI with the type strain was ≥ 95% (the threshold for isDDH is ≥ 70%). Consequently, isolate 520 could be classified into the species *Enterococcus faecium* and was ultimately named *Enterococcus faecium* 520.

The complete genome sequence of *E. faecium* 520 was obtained to explore the molecular characteristics of the isolate. The genome had a 2.436 Mb chromosome encoding 2,284 ORFs and an average GC content of 38.35%, along with a circular plasmid (pEF520–244) consisting of 244,127 bp encoding 253 ORFs (Table 4). Comparative genomic analysis revealed that the chromosome sequence of *E. faecium* SRR24 (2.796 Mb) was most similar to the genome of *E. faecium* 520 (92.73% coverage and 98.71% identity). Analogous to *E. faecium* 520, *E. faecium* SRR24 also possessed a plasmid pSRR24 (CP038997.1). However, its size (123,020 bp) was nearly half that of pEF520–244 (244,127 bp), sharing a low similarity of 13.76 (13.77% coverage and 99.95% identity).

**TABLE 4:** General features of the *E. faecium* 520 genome.

Characteristics	Chromosome	pEF520-244
Size (bp)	2,436,252	244,127
GC content (%)	38.35	35.9
Predicted coding sequences (CDSs)	2,284	253
Known proteins	1,389	124
Hypothetical proteins	895	129
Protein coding (%)	96.37	100
Average ORF length (bp)	887.3	779.1
Average protein length (aa)	301.2	258.7
tRNAs	69	0
rRNA operons	(16S-23S-5S) × 4	0

**FIGURE 1:**
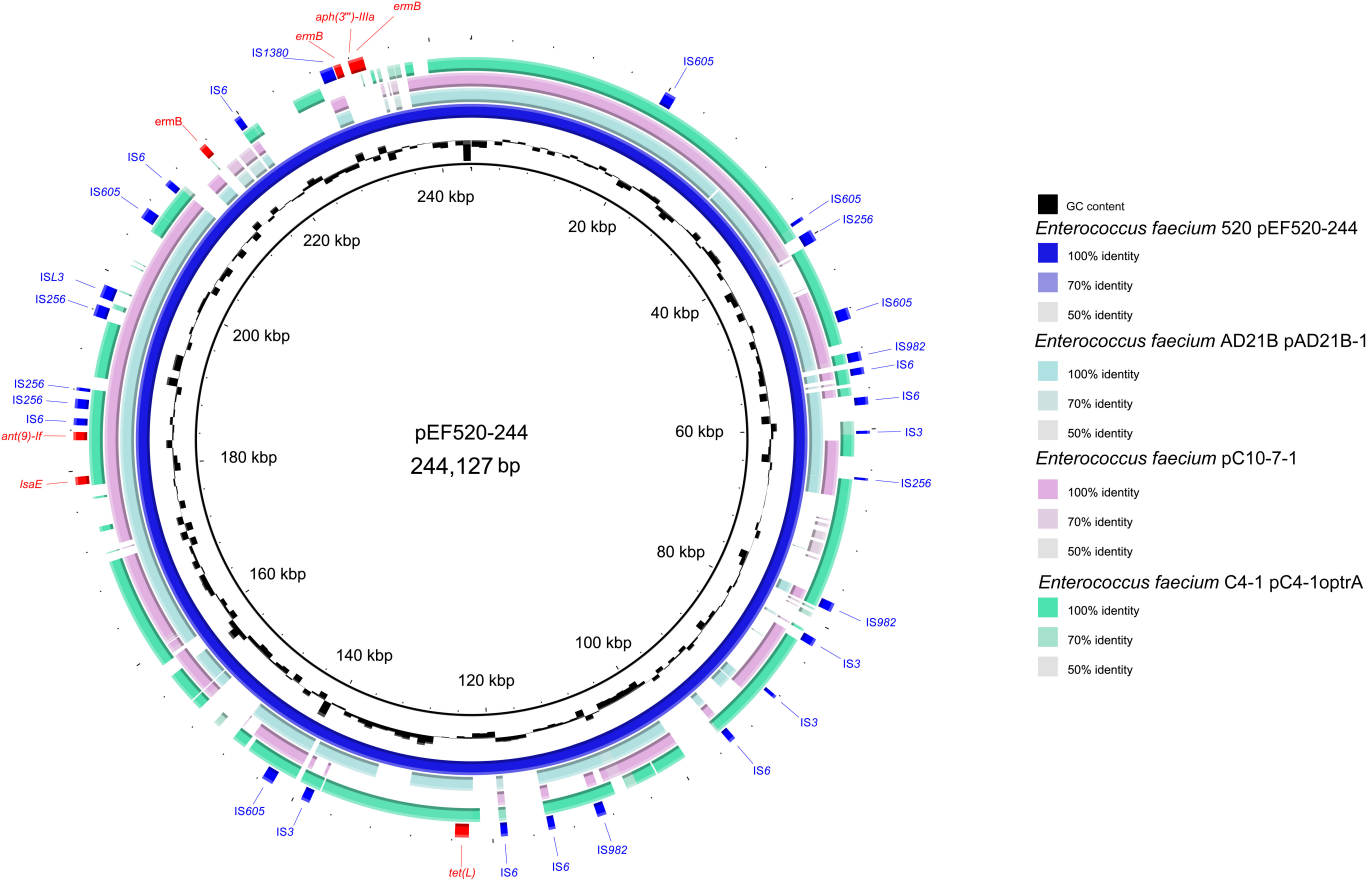
Comparative analysis of pEF520-244 with three other plasmids. Circle 1 (from outer to inner) show the resistance genes and MGEs encoded on the plasmid; circles 2, 3, and 4 show the homologous regions of pC4-1-optrA of *Enterococcus faecium* C4-1, pC10-7-1 of *Enterococcus faecium* and pAD21B-1 of *Enterococcus faecium* AD21B with those of pEF520-244 (circle 5), respectively, and the unmatched regions are left blank. Circles 6 and 7 illustrate the GC content and scale of pEF520-244 in kb.

The functional annotation of the whole genome revealed that it encodes 9 resistance genes that shared ≥ 95.0% similarity with functionally characterized resistance genes, including 2 aminoglycoside (*aac(6’)-Ii* and *aph(3’)-IIIa*), 3 macrolide (3 *ermB*), 1 oxazolidinone (*optrA*), 1 chloramphenicol (*fexA*), 1 tetracycline (*tet(L)*), and 1 lincosamide (*lsaE*) resistance genes. Three genes (*aac(6’)-Ii*, *optrA* and *fexA*) were encoded on the chromosome, whereas the remaining 6, along with the novel resistance gene *ant(9)-If*, were encoded on pEF520–244 (Figure 1).

### Distribution and genetic context of *ant(9)-If*(-like) genes

The novel adenylyltransferase gene *ant(9)-If* was 1,059 bp in length encoding a 352-aa protein with a molecular weight of approximately 20.33 kDa and an isoelectric point of 6.14. A thorough search of the NCBI nucleotide database revealed 232 *ant(9)-If* homologous genes with 100% nucleotide sequence similarity. Excluding one from an isolate of an unclassified species, the remaining 231 genes were derived from 32 species across 9 genera. The genus *Enterococcus* contributed the greatest proportion (58.6%, 136/232), followed by *Streptococcus* (29.3%, 68/232). At the species level, *Enterococcus faecalis* ranked first (37.9%, 88/232), followed by *Enterococcus faecium* (17.2%, 40/232), *Staphylococcus aureus* (10.8%, 25/232), and other species. These bacteria carrying *ant(9)-If*(-like) genes were isolated from diverse sources, including animal and human clinical specimens (Supplementary Table 1). These findings demonstrated that the *ant(9)-If*(-like) genes may spread through close animal–human contact, the food chain and environmental contamination. Such spreading pathways demands the critical need for sustained surveillance of antimicrobial resistance, and reinforce the importance of the One Health approach (De Mesquita Souza Saraiva et al., 2022), which integrates antimicrobial resistance monitoring across humans, animals, and the environment.

**FIGURE 2:**
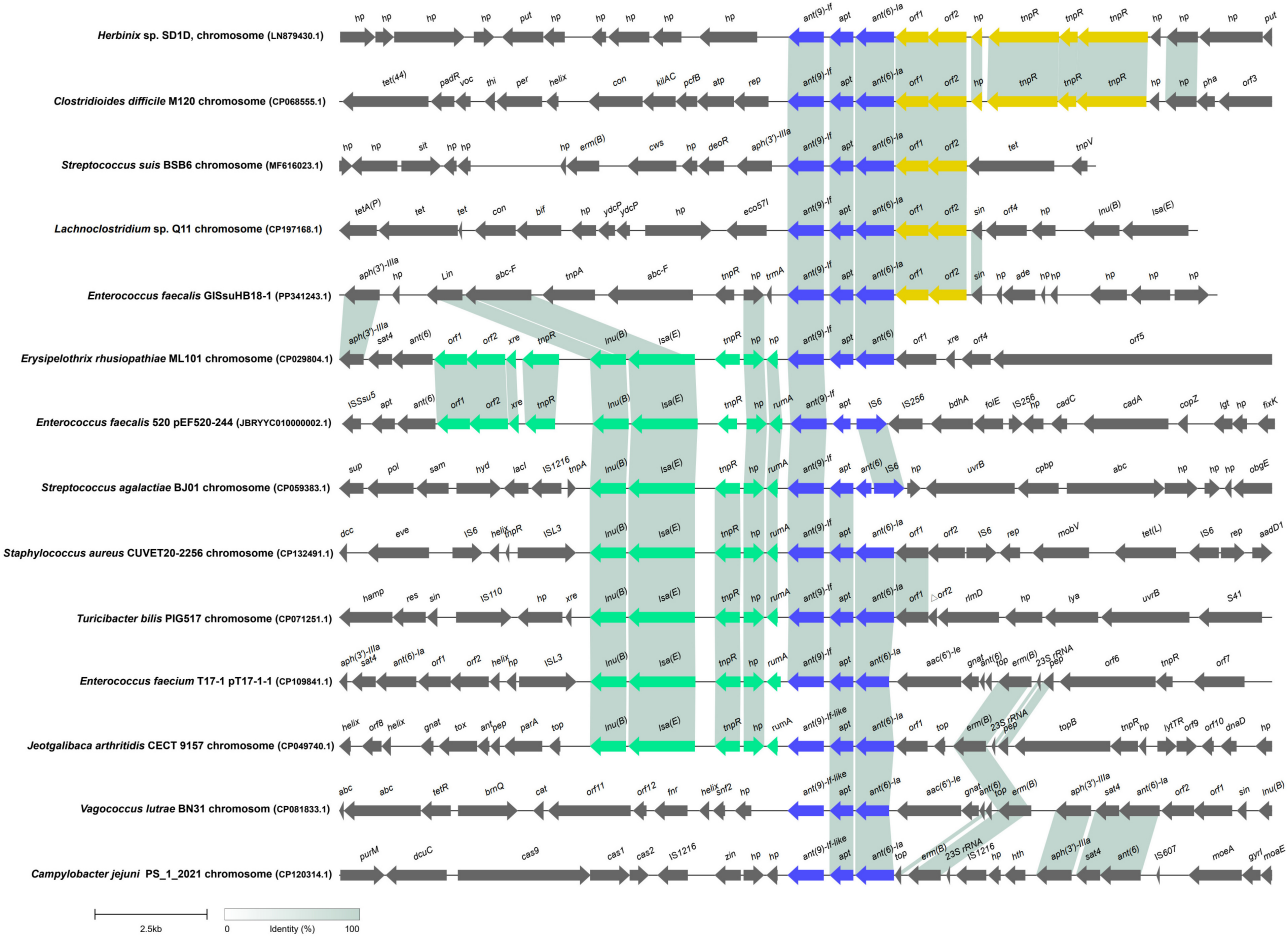
Genetic context of *ant(9)-If* and other related sequences. Homologous genes are represented by the same colors, whereas genes lacking homologs are shown in gray. hp, hypothetical proteins; orf1, class I SAM-dependent methyltransferase; orf2, nucleotidyltransferase domain-containing protein; orf3, glycosyl hydrolase; orf4, restriction endonuclease subunit S; orf4, DNA-binding protein; orf5, DNA helicase; orf6, DNA topoisomerase 3; orf7, MFS transporter; orf8, DUF2207 domain-containing protein; orf9, DUF3021 domain-containing protein; orf10, DUF2812 domain-containing protein; orf11, ATPase; orf12, iron-sulfur cluster repair di-iron protein.

The *ant(9)-If* gene was located on plasmid pEF520-244 of *E. faecium* 520. In contrast, the *ant(9)-If*(-like) genes of the other genera, such as *Enterococcus*, *Listeria*, and *Staphylococcus*, were encoded on both chromosomes and plasmids (Supplementary Table 1). One or two sequences 20 kb in length with *ant(9)-If*(-like) genes at centers from each of the 9 genera were compared to obtain a more in-depth understanding of the genetic environment of the *ant(9)-If*(-like) genes. These 20 kb fragments were generally rich in MGEs and antimicrobial resistance genes. With respect to the sequence reported in this work, the *ant(9)-If* gene, together with two lincosamide resistance genes *lsa(E)* and *lnu(B)*, were associated with two ISs (IS*256* and IS*6*) in a gene array of IS*256*-IS*6*-*apt-2*-*ant(9)*-*rumA*-*hp*-*tnpR*-*lsa(E)*-*lnu(B)*. In contrast to IS*256* and IS*6*, the main part of the sequence [*apt-2*-*ant(9)*-*rumA*-*hp*-*tnpR*-*lsa(E)*-*lnu(B)*] was conserved in most of the other 20 kb sequences illustrated (Figure 2). These findings indicated that the insertion of the IS-related sequence in this study might be associated with the assistance of the IS elements (IS*256* and/or IS*6*). It has been reported that some *ant(9)-I* genes such as *ant(9)-Ia* (CAA26963), *ant(9)-Ib* (AAA16527), *spd* (AGW81558) and *spw* (EGP12870) were also found to be associated with the MGEs. *ant(9)-Ia* was first found on transposon Tn*554* in *Staphylococcus aureus* (Murphy, 1985). The *ant(9)-Ib* gene was identified on a plasmid pDL55 in *Enterococcus faecalis* LDR55 (LeBlanc et al., 1991). Moreover, two additional *ant(9)-I* family members, *spd* and *spw*, were also located on multiresistance plasmids in staphylococci, with *spw* frequently associated with *aadE*, *lnu(B)* and *lsa(E)* in conserved resistance gene clusters (Wendlandt et al., 2013; Jamrozy et al., 2014). However, *ant(9)-Ic* (MZ241296) (Sheng et al., 2023) and *ant(9)-Id* (Yu et al., 2024) were encoded in the chromosomal regions without detectable MGEs.

### Evolutionary and structural relationships of the novel adenylyltransferase ANT(9)-If with functionally characterized proteins

**FIGURE 3:**
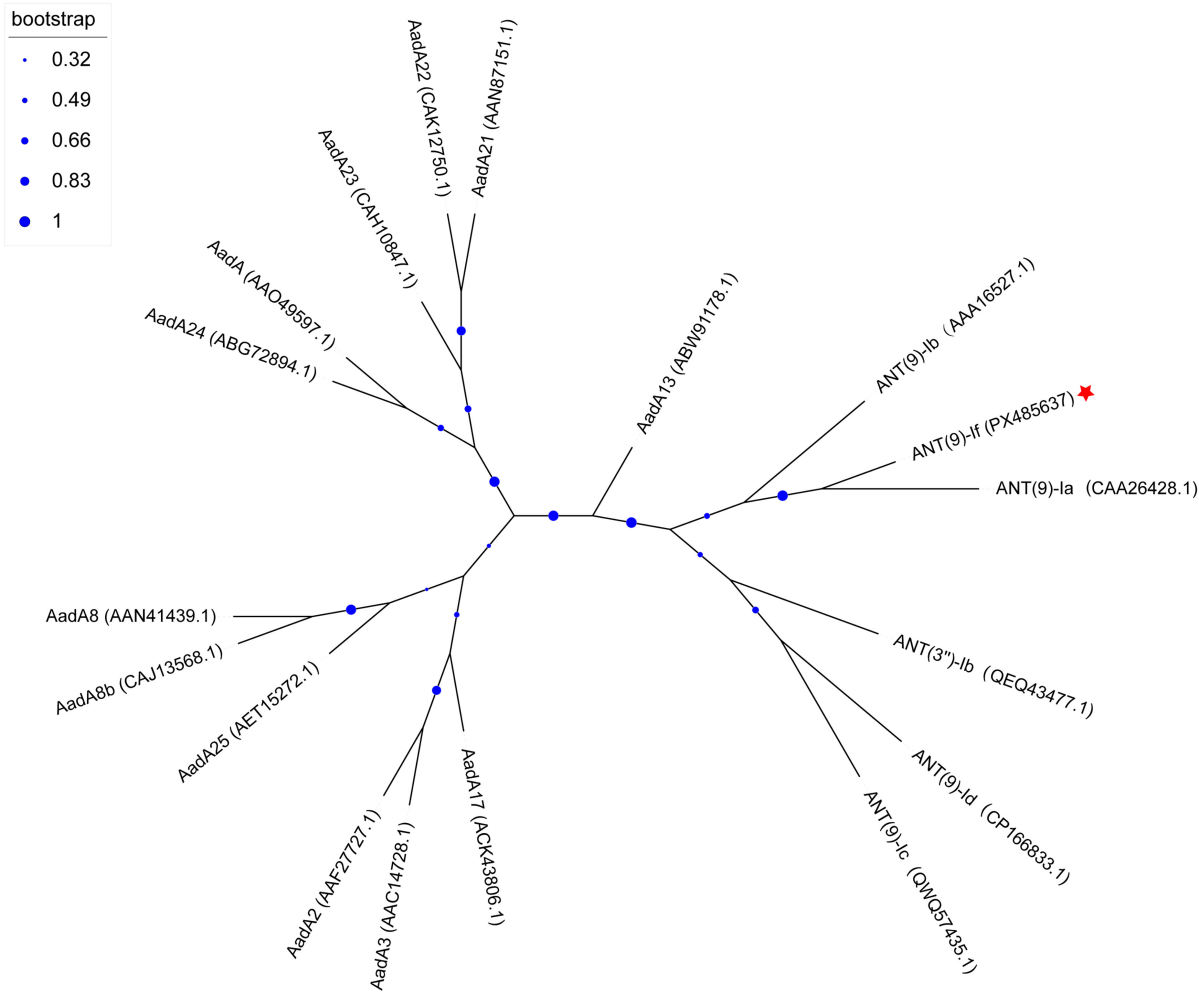
Phylogenetic relationships of ANT(9)-If with other homologous proteins. ANT(9)-If identified in this study is highlighted with a red star.

A total of 17 ANT(9)-If homologous proteins with an identity of ≥ 36.85% to ANT(9)-If were retrieved to analyze the evolutionary relationship between ANT(9)-If and the functionally characterized proteins in the CARD database. These homologous proteins included 5 ANT(9)s (29.41%, 5/17) and 12 ANT(3”)s (70.59%, 12/17) (Figure 3 and Supplementary Table 2). ANT(9)-If exhibited the highest similarity to ANT(9)-Ia (CAA26428.1, 62.98%), followed by AadA (AAO49597.1, 42.50%), ANT(3”)-Ib (QEQ43477.1, 42.18%), and other proteins. The phylogenetic tree indicated that ANT(9)-If closely clustered with the protein ANT(9)-Ia. These findings further supported the possibility that the novel resistance protein identified in this study belonged to the ANT(9)-I family.

**FIGURE 4:**
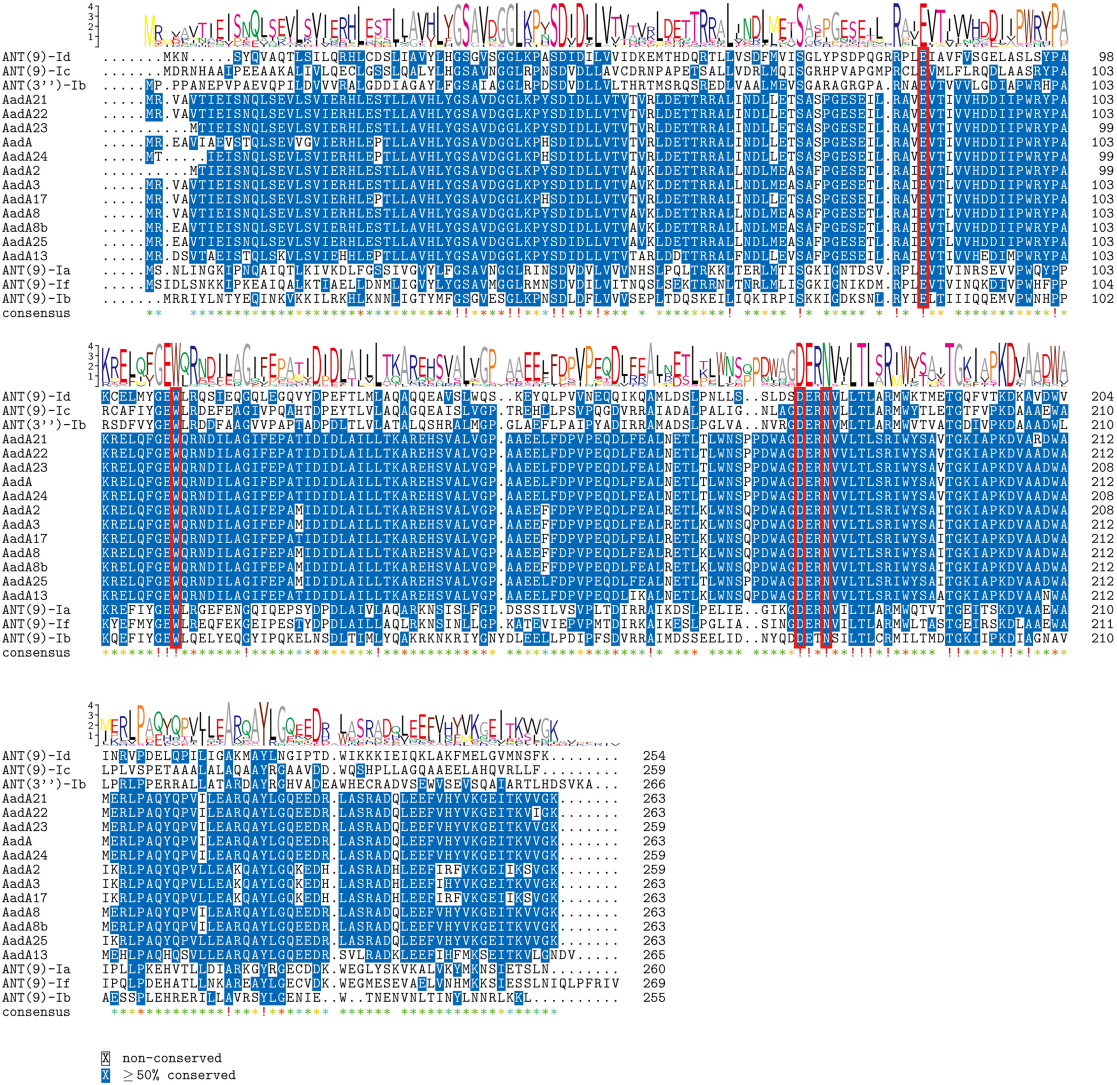
Comparative analysis of aa sequences among ANT(9)-If and its homologous proteins. The numbers on the right indicate the number of aa residues in each full-length protein. Conserved motifs are boxed, with fully conserved residues marked with exclamation marks and highly similar residues denoted with asterisks. The gaps are represented using hyphens. The red frames indicate functional residues. The sequences and their accession numbers are the same as those in Figure 2 and Supplementary Table 2.

Multiple sequence alignment of ANT(9) enzymes was performed to analyze their essential functional residues. The adenylylation of spectinomycin by AadA involves four key amino acid residues: E87, W112, D182, and either H185 or N185 (Stern et al., 2018). These residues are also conserved in ANT(9)-If (E89, W114, D181, and N184), as well as the other ANT(9) proteins (Figure 4). This structural conservation contributes to the similar substrate specificity of the ANT(9)-family enzymes.

### Kinetic parameters of ANT(9)-If

A study of the kinetics of the novel adenylyltransferase ANT(9)-If revealed its ability to adenylylate spectinomycin, which was consistent with the resistance function of the *ant(9)-If* gene. The Michaelis–Menten constant (*K*_*m*_) of the enzyme was 4.599 ± 0.198 μM, and the catalytic efficiency (*k*_*cat*_/*K*_*m*_) was 8.78 ± 0.39 × 10^4^ M^–1^⋅s^–1^. Compared with previously reported ANT(9)-I enzymes, ANT(9)-If demonstrated the highest affinity (*K*_*m*_). However, the catalytic efficiency (*k*_*cat*_/*K*_*m*_) was similar to that of ANT(9)-Ib but approximately 3- and 3,000-fold higher than that of ANT(9)-Ic and ANT(9)-Id, respectively (Table 5).

**TABLE 5:** Kinetic parameters of the ANT(9)-I proteins.

Substrate	*k*_*cat*_	*K*_*m*_ (μ M)	*k*_*cat*_/*K*_*m*_ (M^–1^⋅s^–1^)	Reference
ANT(9)-Ib	2.6 ± 0.2	33.56 ± 8.1	(8.3 ± 2.1) × 10^4^	(LeBlanc et al., 1991)
ANT(9)-Ic	1.2 ± 0.4	44.83 ± 6.2	(2.8 ± 0.6) × 10^4^	(Sheng et al., 2023)
ANT(9)-Id	231.57 ± 59.8	8.94 ± 2.5	26.15 ± 2.95	(Yu et al., 2024)
ANT(9)-If	0.4038 ± 0.00475	4.599 ± 0.198	(8.78 ± 0.39) × 10^4^	This work

### Conclusion

In this study, a new aminoglycoside adenylyltransferase gene, *ant(9)-If*, was identified in an *E. faecium* strain that was resistant to spectinomycin, and the protein it encodes had the highest amino acid similarity to ANT(9)-Ia. Often encoded on plasmids, *ant(9)-If*-like genes are typically associated with mobile genetic elements (MGEs) and are predominantly present in Gram-positive cocci and bacilli across various bacterial species. There are several limitations of this work. To determine the distribution of *ant(9)-If*, large-scale screening of *Enterococcus* strains from various sources should be conducted. To study the transfer ability of the *ant(9)-If gene-*carrying plasmid, conjugation experiments must be performed, and the potential fitness cost of the host carrying the resistance plasmid pEF520-244 should be further studied. Identifying a novel resistance gene and characterizing its biological features would improve our understanding of the complexity of bacterial antimicrobial resistance mechanisms.

## Data Availability

The datasets presented in this study can be found in online repositories. The names of the repository/repositories and accession number(s) can be found in the article/Supplementary material.

## Supplementary material

Supplementary Table 1 Distribution and sources of the *ant(9)-If*(-like) genes.

Supplementary Table 2 Functionally characterized proteins with close homologous relationships with ANT(9)-If.
